# A glial oxidative signature predicts disability in primary progressive multiple sclerosis and is associated with long-term cognitive decline

**DOI:** 10.3389/fimmu.2026.1802339

**Published:** 2026-04-27

**Authors:** Albert Miguela, Joana Maria Huertas-Pons, Clàudia Coll-Martinez, Ariadna Gifreu-Fraixinó, Judit Salavedra-Pont, Manuel Comabella, Luisa María Villar, Jordi Gich, Gary Álvarez-Bravo, Lluís Ramió-Torrentà, Ana Quiroga-Varela

**Affiliations:** 1 Neurodegeneration and Neuroinflammation Group, Girona Biomedical Research Institute (IDIBGI-CERCA), Salt, Spain; 2 Red de Enfermedades Inflamatorias, Carlos III Health Institute (ISCIII), Madrid, Spain; 3 Department of Neurology, Girona Neuroimmunology and Multiple Sclerosis Unit, Dr. Josep Trueta University Hospital and Santa Caterina Hospital, Girona/Salt, Spain; 4 Servei de Neurologia, Centre d’Esclerosi Múltiple de Catalunya (Cemcat), Institut de Recerca Vall d’Hebron (VHIR), Hospital Universitari Vall d’Hebron, Universitat Autònoma de Barcelona, Barcelona, Spain; 5 Center for Networked Biomedical Research on Neurodegenerative Diseases (CIBERNED) - Carlos III Health Institute (ISCIII), Madrid, Spain; 6 Department of Immunology, Hospital Ramón y Cajal, Ramon y Cajal Institute for Health Research (IRYCIS), Spain, Madrid, Spain; 7 Department of Medical Sciences, Faculty of Medicine, University of Girona, Girona, Spain; 8 Medical Management Department, Fundació Hospital d’Olot i Comarcal de la Garrotxa, Olot, Spain

**Keywords:** biomarkers, cognition, cytokines, multiple sclerosis, neurodegeneration, oxidative stress, progression

## Abstract

**Background:**

Progressive multiple sclerosis (MS) involves heterogeneous mechanisms, and primary progressive MS without inflammatory activity (PPMS-NA) is poorly understood.

**Objective:**

To characterize the immune–oxidative profile of PPMS-NA and assess plasma IL-6, IL-8, IFNα2, and cerebrospinal fluid (CSF) reactive oxygen species (ROS) as predictors of disability and cognitive decline.

**Methods:**

We conducted a multicenter longitudinal study with two cohorts. Cohort 1 included participants across MS phenotypes (n = 146 baseline; n = 36 follow-up) and other neurological disorders, measuring plasma cytokines and CSF ROS. Cohort 2 included 40 people with MS, followed for 10 years to assess associations with cognition. Outcomes were analyzed with multivariate models.

**Results:**

PPMS-NA showed reduced IL-6 and IFNα2 but elevated CSF ROS versus RRMS and OND, reflecting low systemic inflammation and central oxidative stress. IL-8 increased, predicting disability and 10-year processing speed decline. IL-6 had time- and domain-specific effects: initially linked to attention, later inversely with visuospatial and working memory. CSF ROS correlated with atrophy, supporting oxidative stress as neurodegeneration driver.

**Conclusion:**

PPMS-NA is a progressive MS subtype driven by oxidative and glial processes. IL-8 and CSF ROS are biomarkers for early stratification, and IL-6 relates to cognition, supporting therapies targeting oxidative stress and glia.

## 1 Introduction

Multiple sclerosis (MS) is a chronic, neurodegenerative, and immune-mediated disease of the central nervous system (CNS) characterized by demyelination and axonal damage. It manifests as relapsing-remitting MS (RRMS), secondary progressive MS (SPMS), and primary progressive MS (PPMS). While RRMS is characterized by acute, episodic inflammation, both PPMS and SPMS exhibit progressive neurological decline. SPMS typically follows a relapsing-remitting course, whereas PPMS progresses from onset (1–3).

PPMS is increasingly recognized as a heterogeneous entity and can be subclassified into active (PPMS-A) and non-active (PPMS-NA) forms. PPMS-A involves ongoing inflammatory activity, evidenced by relapses or radiologically by gadolinium-enhancing lesions (Gd+) or new/enlarging T2 lesions on MRI (4, 5). PPMS-NA refers to progressive disability without inflammatory activity, primarily driven by neurodegenerative processes (3).

Chronic microglial activation underlies the pathophysiology of PPMS-NA (6). Activated microglia and astrocytes release pro-inflammatory cytokines: interleukin-6 (IL-6) and interleukin-8 (IL-8), promoting axonal damage and impaired remyelination (7, 8). IFNα2 sustains innate immune activation (9–11), whereas reactive oxygen species (ROS), largely microglial-derived, drive oxidative stress, excitotoxicity, mitochondrial dysfunction, and neuronal loss (12, 13). These immune–oxidative processes persist in PPMS-NA, contributing to subclinical neurodegeneration (14) and potential biomarkers of disease progression. Elevated CSF cytokines and oxidative stress markers have been linked to greater atrophy and disability (15, 16), and may contribute to early cognitive decline, affecting attention, memory, and processing speed (1, 17). Studying cytokine and ROS levels may provide critical insight into neurodegeneration mechanisms and help identify early biomarkers.

Our study investigates the role of pro-inflammatory cytokines (IFNα2, IL-6, and IL-8) and ROS in PPMS-NA pathogenesis, particularly focusing on their association with neurodegeneration and cognitive decline. By exploring their relationship with clinical features, we aim to identify biomarkers to guide diagnosis and therapy in this MS subtype.

## 2 Materials and methods

### 2.1 Study design and participants

This multicenter retrospective longitudinal study included 146 adults with multiple sclerosis (pwMS) or other neurological disorders (OND), recruited from the Girona Neuroimmunology and Multiple Sclerosis Unit at Dr. Josep Trueta University Hospital and Santa Caterina Hospital (Girona-Salt, Spain), the Multiple Sclerosis Centre of Catalonia at Vall d’Hebron University Hospital (Barcelona, Spain), and the Immunology Department at Ramón y Cajal University Hospital (Madrid, Spain). OND served as controls to distinguish MS-specific changes in cytokines and oxidative biomarkers. A subset (N = 36) had longitudinal follow-up, and an independent pwMS cohort (N = 40) underwent cognitive assessments at baseline, 5, and 10 years. Analyses included participants with complete clinical and biomarker data; longitudinal analyses were limited by follow-up availability.

Participants were ≥18 years and diagnosed with MS per the 2017 revised McDonald criteria (18) or classified as OND with inflammatory (OND-I) or non-inflammatory (OND-NI) profiles (**Supplementary Table S1**). pwMS were categorized into RRMS (relapses with recovery), SPMS (progressive decline following RRMS history), or PPMS (continuous progression from onset for ≥1 year without relapses), further subclassified into PPMS-NA and PPMS-A. PPMS-NA was defined by the absence of relapses, Gd+ lesions, and new T2 lesions over 3 years. Exclusion criteria included other neurodegenerative diseases, traumatic brain injury, psychiatric disorders, substance abuse, or corticosteroid treatment at sample collection.

The study was approved by the Ethics Committee from Dr. Josep Trueta University Hospital (approval numbers: 2011.016 and 2023.206). Written informed consent was obtained from all participants for the collection and use of demographic, clinical data, and biomarker data, as well as biological samples, in accordance with the Declaration of Helsinki, relevant data protection regulations, and IDIBGI biobank guidelines (B.0000872; consent codes: 007/2014 and 008/2014).

### 2.2 Biological samples and data collection

Blood samples were collected at baseline and follow-up, with SPMS samples timed near the confirmed conversion. Plasma was isolated via centrifugation (2000g, 15 min) from EDTA-treated tubes (BD, Franklin Lakes, NJ, USA) and stored at −80 °C for cytokine analysis. CSF samples for ROS analysis were obtained at diagnosis, centrifuged (400g, 15 min), and stored similarly.

Baseline data included demographic, clinical disability (measured with the Expanded Disability Status Scale; EDSS). MRI outputs included visible brain atrophy (presence/absence) as a proxy for neurodegeneration, Gd-enhancing T1 lesions (present/absent), and T2 lesion burden (categorized in: 0–9, 10–50, >50 lesions). Scans were typically performed within six months of sampling. Follow-up variables included disease duration (from symptom onset to sampling), EDSS, and disease-modifying therapy (DMT) classified as: no treatment, moderately effective (dimethyl fumarate [n=2], teriflunomide [n=5]), and highly effective (rituximab [n=8], ocrelizumab [n=6], cladribine, ozanimod, natalizumab, BTK inhibitor [n=1 each]).

### 2.3 Cytokines multiplex assay

Plasma cytokine and chemokine levels (GM-CSF, IFNα2, IL-1Ra, IL-1β, IL-6, IL-8, IP-10) were measured using a customized MILLIPLEX Panel A (HCYTA-60K, Millipore Sigma, Burlington, MA, USA), based on magnetic bead-based antibody detection and processed per the manufacturer’s protocol. Fluorescence was read on a Luminex MAGPIX instrument (Software xPONENT v4.3) and analyzed with Belysa Immunoassay Software (MilliporeSigma, Burlington, MA, USA). Samples were run in duplicate; undetectable or out-of-range values were replaced with the lowest or highest standard, respectively.

### 2.4 Determination of ROS levels in CSF

Oxidative stress in the CSF of pwMS and OND was quantified by measuring the concentration of hydrogen peroxide (H_2_O_2_), a type of ROS. H_2_O_2_ levels were determined using the ROS-Glo H_2_O_2_ Assay (Promega, Madison, WI, USA), following the manufacturer’s instructions. Luminescence was measured in Relative Light Units (RLU) using a GloMax 96 Microplate Luminometer (Promega, Madison, WI, USA) and normalized to protein content or volume. All samples were run in duplicate, with blank and positive controls included for quality assurance.

### 2.5 Cognitive and behavioral assessment

Cognitive performance was assessed using the Brief Repeatable Battery of Neuropsychological Tests (BRB-N) (19, 20) which includes the 10/36 Spatial Recall Test (SPART), Symbol Digit Modalities Test (SDMT), Paced Auditory Serial Addition Test (PASAT), Word List Generation Test (WLG), and the Selective Reminding Test (SRT) with its domains: Long-Term Storage (SRT LTS), Consistent Long-Term Retrieval (SRT CLTR), and Delayed Recall (SRT DR), along with the Trail Making Test Parts A and B (TMT-A, TMT-B), which evaluate processing speed and executive function, respectively (21, 22). To account for potential mood-related influences on cognitive performance, anxiety and depression symptoms were evaluated using the Hospital Anxiety and Depression Scale (HADS) (23).

### 2.6 Statistical analysis

Data normality was assessed using the Shapiro–Wilk test. Group comparisons were performed with Student’s t-test or Mann–Whitney U test (two groups) and one-way ANOVA with Bonferroni correction or Kruskal–Wallis with Dunn’s test (≥3 groups). Categorical variables were analyzed with χ² tests. Longitudinal cytokine changes were evaluated using repeated-measures ANOVA or Friedman tests. Multiple comparisons were controlled with Bonferroni and Benjamini–Hochberg FDR corrections. Associations between baseline cytokines and cognitive outcomes were assessed via multivariate linear regression adjusted for age, sex, education, and HADS scores. Baseline DMT exposure was evaluated using non-parametric Kruskal-Wallis tests, which showed no significant relationship with 5 and 10-year cognitive change (all p > 0.05). Consequently, DMTs were not included as covariates in the predictive analyses.

Logistic regression identified predictors of disability progression (EDSS ≥6), reported as Odds Ratios (ORs) or standardized β with 95% CIs. Analyses were adjusted for key confounders, and sensitivity analyses excluded participants with incomplete data. Sex-stratified analyses were performed for all key biomarkers and cognitive outcomes to assess potential confounding effects, with results reported in the **Supplementary Material** (**Supplementary Table S2**). All tests were two-tailed, with p < 0.05 considered significant. Analyses were performed using IBM SPSS Statistics v21 and GraphPad Prism v8.

## 3 Results

### 3.1 Clinical and demographic characteristics

Baseline demographic and clinical characteristics are summarized in **Table 1**. The baseline cohort (n = 146) included RRMS (n= 69; 71% female), PPMS-NA (n= 30; 50% female), PPMS-A (n=10; 40% female), 11 with SPMS (n=11; 81.8% female), and OND (n=26 subdivided into OND-NI with 13 participants, 76.9% female, and OND-I; 13 participants, 61.5% female).

**Table 1:** Demographic, clinical, and radiological characteristics of the study participants at baseline.

Demographics & clinical							
Variable	RRMS	PPMS-NA	PPMS-A	SPMS	OND-NI	OND-I	p-value
N	69	30	10	9	13	13	
Sex^1^							0.076
Male	20 (29.0)	15 (50.0)	6 (60.0)	1 (11.1)	3 (23.1)	5 (38.5)	
Female	49 (71.0)	15 (50.0)	4 (40.0)	8 (88.9)	10 (76.9)	8 (61.5)	
Age^2^	45 (35.0-52.0)	53.5 (47.0-58.3)	56 (50.5-60.0)	52 (44.0-53.5)	47 (40.0-53.0)	43 (38.5-57.0)	<0.001**
EDSS^2^	2	3.5	4.3	4	–	**-**	<0.001**
	(1.5-2.9)	(3.0-4.6)	(2.8-6.0)	(1.8-6.0)			
Radiological measures							
Range of T2 lesions^1^							0.079
0-9	14 (22.2)	8 (26.7)	0 (0.0)	1 (14.3)	-	**-**	
10-50	47 (74.6)	20 (66.7)	8 (80.0)	4 (57.1)	-	**-**	
>50	2 (3.2)	2 (6.7)	2 (20.0)	2 (28.6)	-	**-**	
Gd+ lesions^1^ (presence)	19 (30.6)	0 (0.0)	6 (60.0)	0 (0.0)	-	**-**	<0.001**
Atrophy^1^ (presence)	5 (7.7)	4 (36.4)	3 (33.3%)	1 (14.3)	–	**-**	0.015*

Data are presented as ^1^number (percentage) for categorical variables and ^2^median (Q1-Q3) for continuous variables. Bold values denote statistically significant results (*p < 0.05, ** p < 0.01).

Age differed across groups (*p* < 0.001), with progressive MS phenotypes (PPMS-NA, PPMS-A, SPMS) being older than RRMS or OND groups. EDSS scores were higher in progressive MS phenotypes (*p* < 0.001) while sex distribution was not significant (*p* = 0.076). Radiologically, Gd+ lesions were most frequent in RRMS (30.6%) and PPMS-A (60.0%), and absent in OND (p < 0.001). Brain atrophy was more prevalent in PPMS-NA (36.4%) and PPMS-A (33.3%) than in RRMS and SPMS (7.7% and 14.3%; *p* = 0.015). T2 lesion burden showed a non-significant trend toward higher counts in progressive MS (*p* = 0.079). All patients in the RRMS, PPMS-NA, and PPMS-A groups were newly diagnosed and untreated at baseline. In the SPMS group (n=9), only 6 patients were receiving high-efficacy DMT due to advanced disease.

In the follow-up cohort (n=36; **Table 2**), PPMS-NA patients were older (*p* < 0.05) with longer disease duration (*p* < 0.05) and higher EDSS scores than RRMS (*p* < 0.001). DMT use differed (*p* < 0.001): 83.3% of PPMS-NA were untreated, all PPMS-A patients received highly effective therapy, and RRMS patients received either high (55.6%) or moderate (38.9%) effective DMTs. Radiology reflected these clinical profiles: PPMS-A and RRMS had the highest T2 lesion burden (>50 lesions in 60.0% and 36.5%), while PPMS-NA had the lowest (0–9 lesions in 20.7%; *p* < 0.05). Gd+ lesions were present in PPMS-A (40.0%) and RRMS (31.7%), but absent in PPMS-NA (*p* < 0.001). Brain atrophy frequency was similar across groups (42.9 - 60.0%; *p* = 0.787).

**Table 2:** Demographic, clinical, and radiological characteristics of pwMS at follow-up.

Demographics & clinical				
Variable	RRMS	PPMS-NA	PPMS-A	p-value
N	18	12	6	
Sex^1^				0.358
Male	6 (33.3)	5 (41.6)	4 (33.33)	
Female	12 (66.7)	7 (58.4)	2 (66.7)	
Age^2^	56.5 (51.8 - 64.3)	65.0 (61.3- 70.00)	59.0 (51.3-65.5)	<0.05*
Disease duration (y)^2a^	6 (5.0–7.25)	12.5(7.25–15.50)	5.5 (4.25–14.5)	<0.05*
Follow-up EDSS^2^	3.25 (2.0–4.0)	6.00 (2.0–4.0)	6.00 (5.3–6.9)	<0.001**
DMT total^1^				<0.001**
None	1 (5.6)	10 (83.3)	0 (0)	
Moderate efficacy^b^	7 (38.9)	0 (0)	0 (0)	
High efficacy^c^	10 (55.6)	2 (16.7)	6 (100.0)	
Radiological measures				
Range of T2 lesions^1^				< 0.05*
0-9	1 (1.6%)	6 (20.7%)	0 (0.0%)	
10-50	39 (61.9%)	17 (58.6%)	4 (40.0%)	
>50	23 (36.5%)	6 (20.7%)	6 (60.0%)	
Gd+ lesions^1^ (presence)	20 (31.7)	0(0)	4 (40.0)	<0.001**
Atrophy^1^ (presence)	8 (44.4)	6 (42.9)	6 (60.0)	0.787

Data are presented as ^1^number (percentage) for categorical variables and ^2^median (Q1-Q3) for continuous variables. ^a^Time between first symptom and follow-up. ^b^Moderately effective DMT (n = 7 patients). ^c^ Highly effective DMT (n = 18 patients). Bold values denote statistically significant results (*p < 0.05, **p < 0.01).

### 3.2 Distinct IFNα2, IL-6, and IL-8 profiles in PPMS-NA over time

Plasma levels of seven cytokines and chemokines were initially measured at diagnosis in a subset of patients (**Supplementary Figure S1**). Among these, IFNα2, IL-6, and IL-8 showed the most pronounced differential expression and were selected for further analysis in the full baseline cohort, including SPMS.

**Figure 1:**
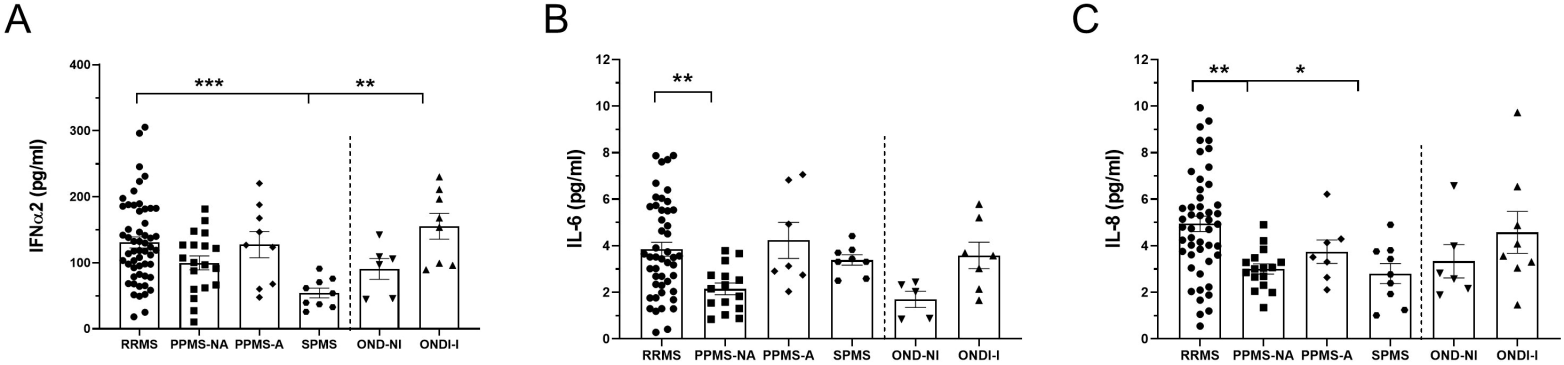
Baseline plasma levels of cytokines IFNα2, IL-6, and IL-8 based on phenotype adjusted by age. Plasma concentration (pg/ml) of IFNα2 **(A)**, IL-6 **(B)**, and IL-8 **(C)** across different MS phenotypes and OND. Data are presented as mean ± SEM, with *p < 0.05; **p < 0.01 and ***p < 0.0001 indicating significant differences. Statistical comparisons for follow-up levels were performed using ANCOVA adjusted for age, followed by Bonferroni-corrected *post hoc* tests.

**Figure 2:**
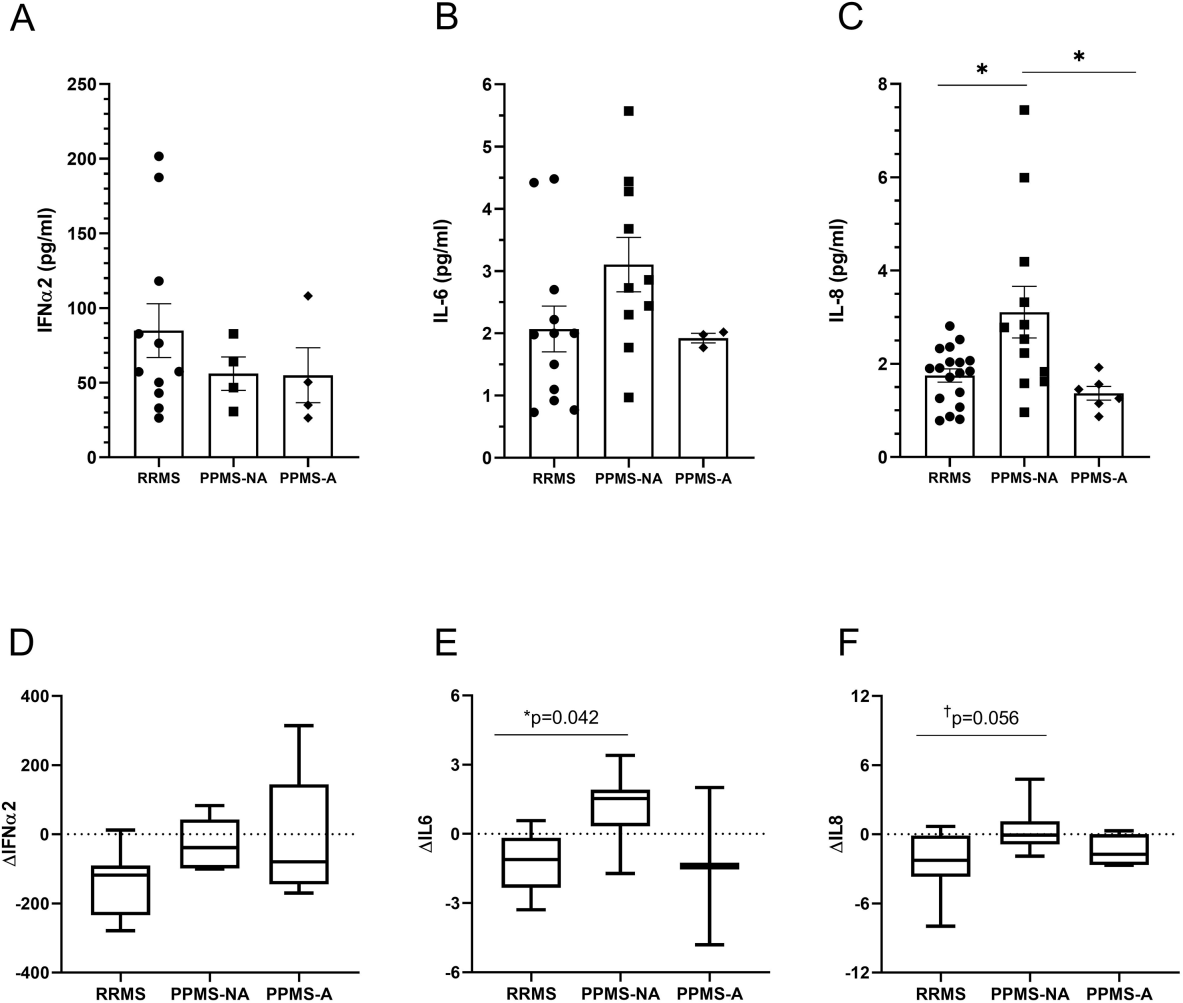
Follow-up plasma levels of cytokines based on phenotype and changes relative to baseline measurement. Plasma concentration (pg/ml) of IFNα2 **(A)**, IL-6 **(B)**, and IL-8 **(C)** at follow-up, and corresponding changes from baseline [Δ; **(D–F)**] across MS phenotypes. Data are shown as mean ± SEM. *p < 0.05; ^†^p = 0.056 indicates trend. Statistical comparisons for follow-up levels were performed using ANCOVA adjusted for age and disease duration, followed by Bonferroni-corrected *post hoc* tests. Δ values were analyzed using Kruskal–Wallis tests and exploratory Wilcoxon pairwise comparisons, with p-values adjusted using FDR correction where appropriate.

At baseline, after adjustment for age, IFNα2 was significantly reduced in the SPMS group compared with RRMS (*p* = 0.0008) and OND-I (*p* = 0.0016; **Figure 1A**). IL-6 was lower in the PPMS-NA group compared with RRMS (*p* = 0.0106; **Figure 1B**), and IL-8 was lower in PPMS-NA (*p* = 0.002) but higher than SPMS (*p* = 0.0155; **Figure 1C**). At follow-up, after adjusting for age and disease duration, IL-8 was significantly higher in PPMS-NA than RRMS (*p* = 0.013) and PPMS-A (*p* = 0.019; **Figures 2A–C**) while IL-6 and IFNα2 showed no significant differences.

Longitudinal changes in cytokine levels (Δ) were assessed for IFNα2, IL-6, and IL-8 (**Figures 2D–F**). Overall differences across MS phenotypes were not statistically significant (ΔIFNα2, *p* = 0.179; ΔIL-6, *p* = 0.095; ΔIL-8, *p* = 0.074). Exploratory pairwise comparisons suggested that PPMS-NA exhibited a smaller reduction in IL-6 than RRMS (*p* = 0.042), with a similar trend for IL-8 (*p* = 0.056); ΔIFNα2 showed no significant change. These findings suggest a blunted anti-inflammatory profile in the PPMS-NA.

No baseline sex differences were observed for any biomarker (all p > 0.1; **Supplementary Table S2**). At follow-up, RRMS females tended toward higher IFNα2 and IL-6 (*p* = 0.0691 and *p* = 0.0872, respectively), while IL-8 was significantly higher in PPMS-A females (*p *= 0.0107). IL-6 could not be compared in PPMS-A at follow-up due to the limited sample size. Overall, these sex-stratified analyses indicate that sex differences are minimal to isolated trends, supporting the robustness of the predictive and comparative analyses adjusted for age and disease duration.

### 3.3 CSF ROS at baseline reveals increased oxidative stress in PPMS-NA

**Figure 3:**
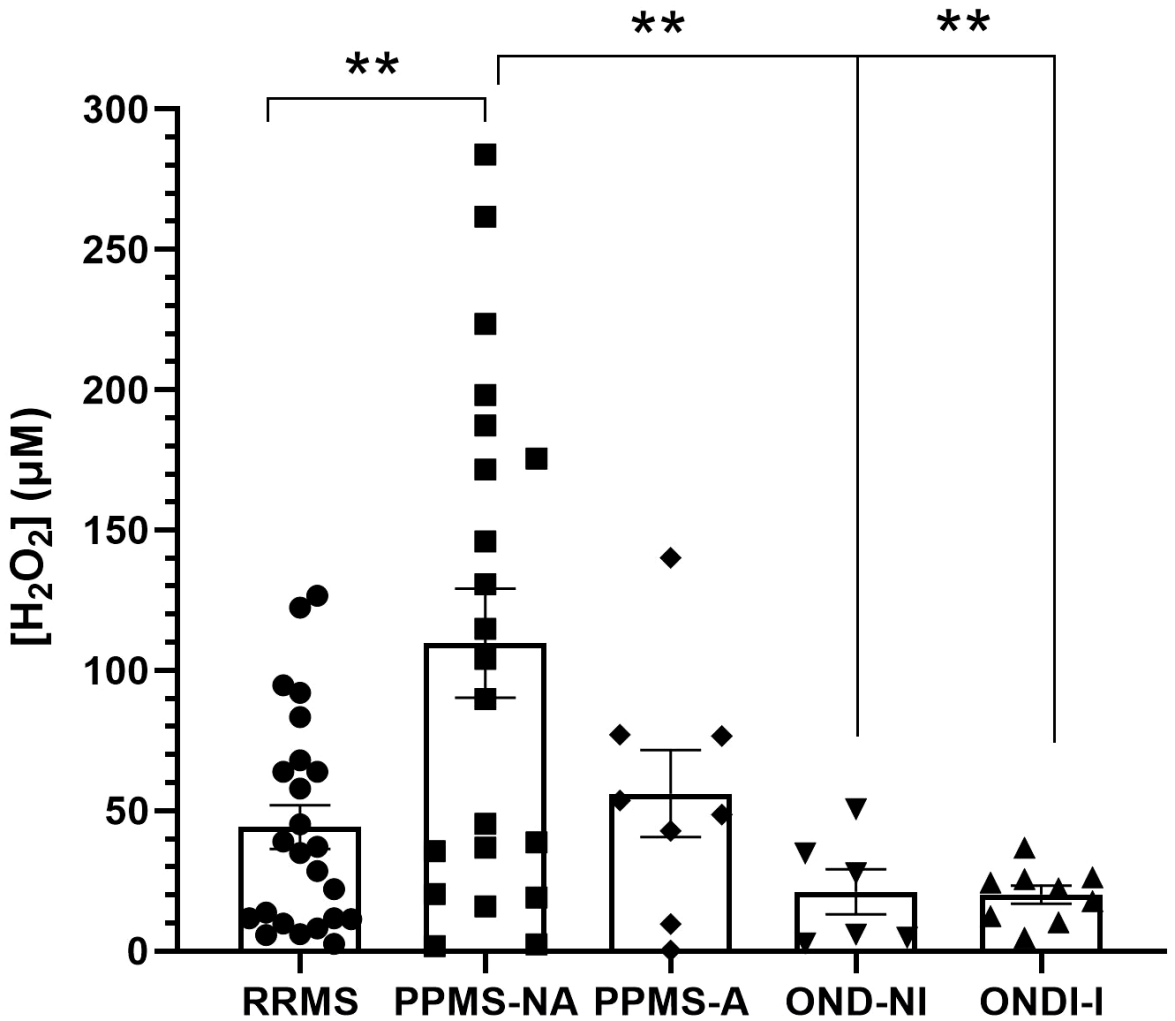
Baseline CSF hydrogen peroxide (H_2_O_2_) levels based on phenotype. H_2_O_2_ concentration (µM) in cerebrospinal fluid across different MS phenotypes. Data are presented as mean ± SEM. **p < 0.01, indicating significant differences relative to PPMS-NA. Statistical comparisons for follow-up levels were performed using ANCOVA adjusted for age, followed by Bonferroni-corrected *post hoc* tests.

CSF H_2_O_2_, used as a proxy for ROS, was higher at baseline in PPMS-NA than in RRMS (*p* = 0.004), OND-I (*p* = 0.0058), and OND-NI (*p* = 0.040; **Figure 3**) after age adjustment. No significant differences were observed between PPMS subtypes (PPMS-A vs. PPMS-NA; p = 0.294), though mean H_2_O_2_ was highest in PPMS-NA (109.4 ± 14.1 vs. PPMS-A 55.9 ± 22.9). Age-adjusted sex analyses showed no significant differences (all p > 0.19), although males tended to have higher H_2_O_2_ than females in PPMS-NA, RRMS, and PPMS-A (See **Supplementary Table S2**).

### 3.4 Baseline IL-8 and CSF H_2_O_2_ associations with MRI-detected atrophy and lesion burden in early MS

We investigated whether baseline plasma cytokines (IFNα2, IL-6, IL-8) and CSF H_2_O_2_ were associated with MRI measures of neurodegeneration and lesion burden: baseline and follow-up atrophy; Gd-enhancing lesions (baseline only due to their marked reduction in pwMS following DMT); and baseline and follow-up T2 lesion load.

No significant associations were observed between baseline plasma cytokines or CSF H_2_O_2_ and baseline atrophy (**Table 3A**). IFNα2 and IL-6 levels were slightly higher in pwMS with atrophy (OR 1.00, *p* = 0.77; OR 0.97, *p* = 0.87), IL-8 showed no association, and CSF H_2_O_2_ trended higher at baseline (OR 3.35, *p* = 0.07) and at follow-up (OR 1.15, *p* = 0.06; **Table 3B**) in those who developed atrophy. These trends suggest that oxidative stress contributes to neurodegenerative progression, whereas systemic cytokines appear less linked.

**Table 3:** Associations of baseline plasma cytokines and CSF H_2_O_2_ with baseline and follow-up MRI outcomes.

A. Biomarkers and their association with MRI-detected atrophy at baseline									
Biomarker (baseline)	Absence of atrophy			Presence of atrophy		OR (95% CI)		P-value	
	Median (IQR)			Median (IQR)					
IFNα2	123.57 (47.64–147.85)			146.57 (91.55–167.59)		1.00 (0.99–1.01)		0.77	
IL-6	2.04 (1.30–3.56)			6.82 (2.74–7.38)		0.97 (0.66–1.42)		0.87	
IL-8	1.61 (0.57)			1.64 (0.60)		2.23 (0.52–9.45)		0.28	
H_2_O_2_	3.64 (1.90)			4.23 (0.76)		3.35 (0.90–12.44)		**0.07†**	
B. Biomarkers and their association with MRI-detected atrophy at follow-up									
Biomarker (baseline)	Absence of atrophy			Presence of atrophy		OR (95% CI)		P-value	
	Median (IQR)			Median (IQR)					
IFNα2	103.32 (75.06–131.14)			131.14 (83.82–180.00)		1.00 (0.99–1.01)		0.38	
IL-6	3.50 (3.36–6.86)			2.74 (1.26–5.50)		0.82 (0.60–1.12)		0.21	
IL-8	1.74 (0.72–2.41)			1.62 (0.35–2.77)		1.71 (0.40–7.30)		0.47	
H_2_O_2_	3.68 (2.04–5.24)			3.95 (1.56–5.43)		1.15 (0.68–1.97)		**0.06†**	
C. Biomarker profiles and Gd-enhancing lesion detection at baseline									
Biomarker (baseline)	Gd^–^			Gd^+^		OR (95% CI)		P-value	
	Median (IQR)			Median (IQR)					
IFNα2	105.35 (74.92–135.32)			135.32 (67.65–180.00)		1.01 (0.99–1.01)		0.22	
IL-6	3.02 (1.81–5.83)			3.02 (2.91–6.83)		1.16 (0.86–1.57)		0.32	
IL-8	1.53 (0.49–2.33)			1.66 (0.40–5.83)		1.82 (0.57–5.83)		0.31	
H_2_O_2_	3.92 (1.87–5.84)			3.87 (1.53–1.53)		0.95 (0.58–1.54)		0.83	
D. Baseline biomarkers and their association with T2 lesion burden at baseline									
Biomarker (baseline)	Low	Moderate	High	Moderate vs Low	95% CI	P-value	High vs Low	95% CI	P-value
	Median (IQR)	Median (IQR)	Median (IQR)	β coefficient			β coefficient		
IFNa2	114.0 (90.1–143.9)	124.0 (89.3–158.4)	61.7 (55.8–67.6)	–12.38	(–60.86, 27.74)	0.513	–52.59	(–118.31, 8.22)	**0.085†**
IL-6	4.36 (2.33–6.47)	3.15 (2.19–4.29)	2.81 (2.09–3.30)	–0.97	(–2.17, 0.23)	0.115	–1.71	(–3.85, 0.42)	0.118
IL-8	5.11 (3.52–6.68)	3.92 (2.70–5.14)	2.71 (2.45–2.96)	–1.14	(–2.53, 0.25)	0.106	–2.48	(–4.91, –0.05)	0.046*
H_2_O_2_	83.4 (2.45–2.96)	36.4 (3.6–69.3)	20.7 (1.6–58.8)	–16.56	(–60.00, 26.88)	0.452	–55.04	(–118.00, 7.92)	**0.086†**
E. Baseline biomarkers and their association with T2 lesion burden at follow-up									
Biomarker (baseline)	Low	Moderate	High	Moderate vs Low	95% CI	P-value	High vs Low	95% CI	P-value
	Median (IQR)	Median (IQR)	Median (IQR)	β coefficient			β coefficient		
IFNa2	122.0 (122.0–122.0)	121.0 (75.0–167.0)	97.8 (60.5–135.2)	12.56	(–60.63, 69.16)	0.843	–9.30	(–85.46, 47.89)	0.883
IL-6	2.52 (2.52–2.52)	3.23 (1.89–5.91)	3.07 (2.25–4.71)	0.72	(–2.58, 4.02)	0.715	0.49	(–3.27, 4.25)	0.803
IL-8	2.85 (2.85–2.85)	4.19 (2.98–6.61)	3.68 (2.60–5.84)	0.94	(–3.01, 4.89)	0.642	0.005	(–4.00, 4.01)	0.998
H_2_O_2_	38.8 (3.8–73.8)	48.6 (10.1–83.1)	37.2 (13.1–62.3)	4.27	(–58.00, 66.54)	0.894	–18.79	(–84.00, 46.42)	0.569

Baseline plasma biomarkers (IFNα2, IL-6, IL-8; pg/mL) and CSF H_2_O_2_ (µM, log-transformed) were analyzed in relation to MRI measures. Logistic regression models were applied for binary outcomes (atrophy at baseline and follow-up, and Gd-enhancing lesions), and linear regression models for ordinal outcomes (T2 lesion burden), categorized as low (<10 lesions), moderate (10–50 lesions), and high (>50 lesions). Baseline MRI analyses were adjusted for age, whereas follow-up analyses were adjusted for age and disease duration. Data are presented as median (IQR). For binary outcomes, odds ratios (OR) with 95% confidence intervals (CI) are reported; for T2 lesion burden, effect sizes are reported as β coefficients with 95% CI. P-values correspond to model estimates and were adjusted for multiple comparisons using the Bonferroni or FDR correction as appropriate. † indicates a trend toward significance (*p* < 0.10), and * indicates statistical significance (*p* < 0.05 after correction). Bold values are used to highlight these statistically significant results.

No significant associations were observed between baseline biomarkers and Gd^+^ lesions (**Table 3C**), with IFNα2, IL-6, IL-8, and CSF H_2_O_2_ levels similar between groups, suggesting that systemic cytokine and CSF oxidative stress levels are independent of acute inflammatory activity.

Baseline biomarkers showed limited associations with T2 lesion burden (**Table 3D**). IL-8 was inversely associated with high versus low lesion load (β = −2.48, *p* = 0.046), while IFNα2 and CSF H_2_O_2_ showed non-significant inverse trends (*p* = 0.085 and *p* = 0.086, respectively). At follow-up (**Table 3E**), no significant associations were observed, with only CSF H_2_O_2_ trending lower levels with higher lesion burden (β = −18.79, *p* = 0.569).

### 3.5 Baseline IL-8, IL-6, and CSF H_2_O_2_ predict future high disability in pwMS

**Figure 4:**
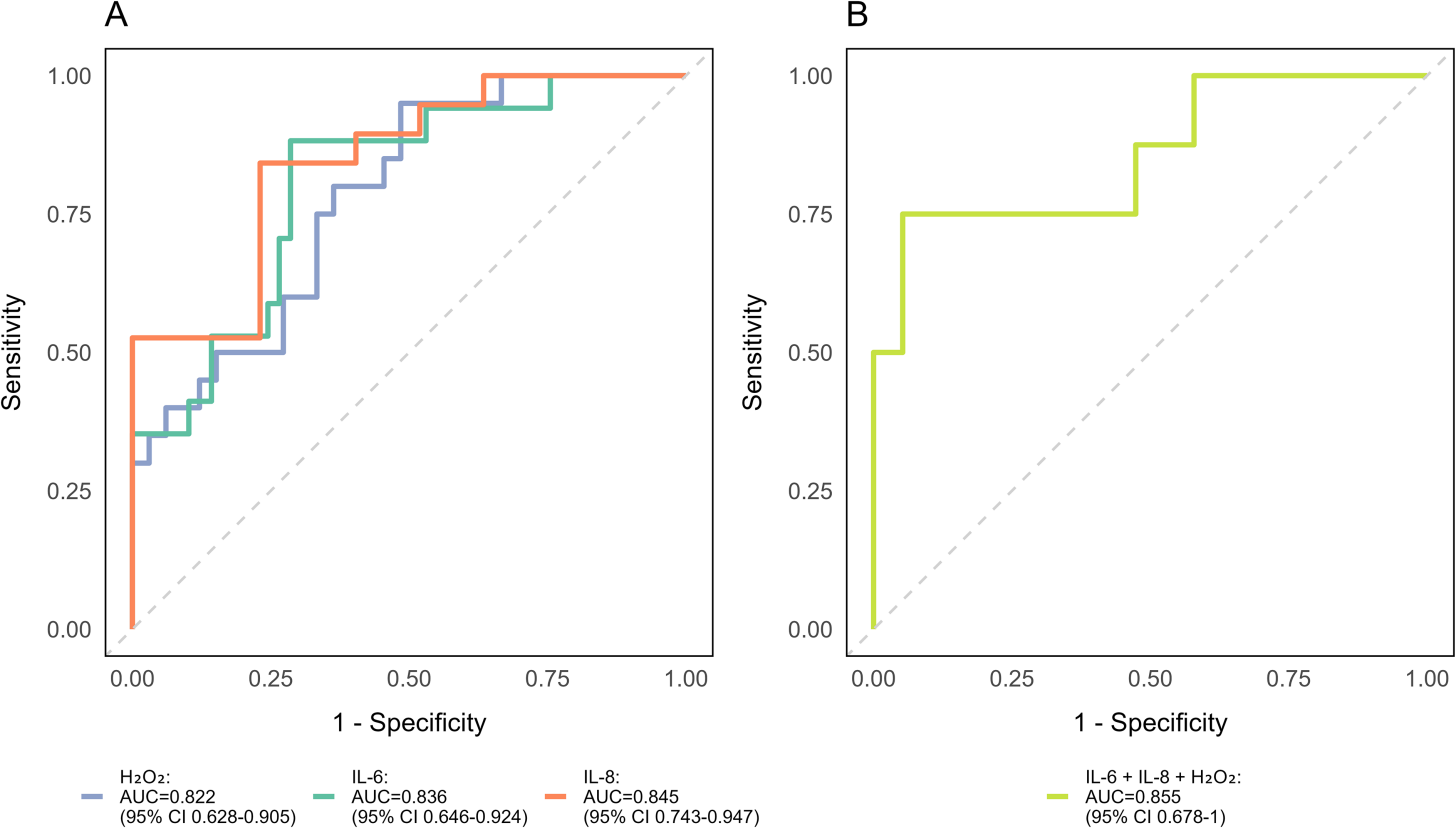
Baseline biomarkers predicting high disability (EDSS ≥6) at follow-up. ROC curves illustrate the discriminative ability of baseline IL-6, IL-8, and CSF H_2_O_2_ for high disability using logistic regression models adjusted for age, baseline EDSS, follow-up duration, and DMT treatment. AUC values and 95% confidence intervals are shown in the graphs. **(A)** Individual biomarkers: IL-8 exhibited the highest discriminative ability, followed by IL-6 and H_2_O_2_. **(B)** Combined model including all three biomarkers, showing a modest improvement over individual markers. P-values were adjusted using FDR correction to account for multiple model comparisons.

Baseline plasma cytokines (IL-6, IL-8) and CSF H_2_O_2_ were evaluated as predictors of higher disability (EDSS ≥6) using logistic regression adjusted for key confounders (**Figure 4A**). IL-8 had the highest predictive ability (AUC = 0.855), followed by IL-6 (AUC = 0.836) and H_2_O_2_ (AUC = 0.822). A combined model slightly increased the AUC (0.855; **Figure 4B**), indicating that IL-8 largely drives predictive performance. These results show IL-8 is the strongest individual predictor, with IL-6 and H_2_O_2_ contributing incrementally.

### 3.6 Baseline IL-8 and IL-6 levels predict mid- and long-term cognitive decline in pwMS

To evaluate IL-6 and IL-8 as predictors of cognitive decline, we analyzed pwMS with cognitive and biomarker data at diagnosis, 5, and 10 years (See **Supplementary Table S3** for demographic and clinical characteristics). Analyses were adjusted for age, education level, and HADS scores.

Baseline IL-8 was not significantly associated with attention, visuospatial or verbal memory, working memory, or verbal fluency (**Table 4**, top). However, higher baseline IL-8 levels significantly predicted reduced processing speed at 5 years (SDMT: β = –0.375, *p* = 0.009), with a trend toward a similar association at 10 years (β = -3.005, *p* = 0.096).

**Table 4:** Multiple linear regression analysis examining the predictive value of baseline IL-8 (top) and IL-6 (bottom) levels on raw scores of cognitive performances.

IL8	Baseline			5-year follow-up			10-year follow-up		
	β-coefficient	(95% CI)	P value	β-coefficient	(95% CI)	P value	β-coefficient	(95% CI)	P value
TMT-A	0,399	(0.38, -2.69)	0,793	1,163	(-1.20, 3.52)	0,320	0,364	(-11.39, 12.12)	0,942
TMT-B	6,012	(6.01, -2.48)	0,158	4,824	(-1.99, 11.60)	0,155	6,836	(-13.69, 27.36)	0,431
SRT LTS	-1,97	(-4,86, 0,92)	0,173	-0,718	(-3,28, 1,84)	0,569	-3,51	(-14,3, 7,26)	0,440
SRT CLTR	-2,61	(-5,39, 0,16)	0,064	-0,463	(-4,02, 3,10)	0,791	-1,43	(-11,1, 8,28)	0,720
SRT DR	-0,314	(-0,78, 0,15)	0,180	-0,106	(-0,62, 0,41)	0,675	-0,859	(-3,03, 1,31)	0,356
SPART	0,655	(0.65, -0.38)	0,209	-0,335	(-1.83, 1.16)	0,649	-2,690	(-3.24, 8.62)	0,310
SDMT	-0,580	(-0.58, -2.90)	0,613	-0,375	(-6.33, -1.18)	0,009**	-3,005	(-7.13, 13.14)	†**0,096**
PASAT	0,056	(0.05, -3.01)	0,970	-0,238	(-4.02, 1.18)	0,269	-6,234	(-6.66, 19.13)	0,282
WLG	-0,723	(-0.72, -2.15)	0,309	-0,099	(-2.02, 1.24)	0,631	-0,786	(-5.96, 7.53)	0,785
IL6	Baseline			5-year follow-up			10-year follow-up		
	β-coefficient	(95% CI)	P value	β-coefficient	(95% CI)	P value	β-coefficient	(95% CI)	P value
TMT-A	0,360	(-0.022, 4.163)	0,050*	0,996	(-0.39, 2.38)	†**0,052**	0,568	(0.13,0.10)	0,020*
TMT-B	-1,323	(-6.934, 4.287)	0,634	2,506	(-1.65, 6.67)	0,227	0,010	(-0.02, 0.04)	0,444
SRT LTS	0,912	(-1,03, 2,85)	0,344	-0,133	(-1,89, 1,63)	0,878	1,07	(-10,4, 12,6)	0,820
SRT CLTR	0,802	(-1,21, 2,81)	0,421	-0,191	(-2,43, 2,05)	0,863	0,139	(-11,9, 12,2)	0,978
SRT DR	0,119	(-0,202, 0,439)	0,454	-0,0472	(-0,41, 0,32)	0,793	-0,0701	(-2,15, 2,01)	0,934
SPART	0,059	(-0.794, 0.911)	0,890	0,046	(-0.87, 0.96)	0,919	-0,109	(-0.23, 0.01)	**0,073†**
SDMT	0,107	(-1.401, 1.614)	0,886	-0,731	(-2.48, 1.02)	0,401	-5,814	(-13.93, 2.30)	**0,081**
PASAT	0,091	(-1.832, 2.015)	0,923	-1,591	(-3.152, -0.03)	0,046*	-0,906	(-0.11, 0.001)	0,043*
WLG	-0,072	(-1.067, 0.923)	0,884	-0,193	(-1.24, 0.86)	0,711	-0,066	(-0.16, 0.16)	0,399

Only results for the biomarker of interest are shown. Age, education, and HADS scores were included as co-variables in each model. Results are presented as β coefficients, 95% confidence intervals (CI), and corresponding p-values. Significance thresholds are indicated as follows: *p < 0.05; **p < 0.01; †*p* < 0.1 (trend). Bold values are used to highlight these statistically significant results.

Baseline IL-6 (**Table 4**, down) showed a borderline significant positive association with attention at baseline (TMT-A: β = 0.360, *p* = 0.050), which persisted as a trend at 5 years (β = 0.996, *p* = 0.052) and reached significance at 10 years (β = 0.568, *p* = 0.020). Conversely, IL-6 tended to negatively associate with visuospatial memory at 10 years (SPART: β = –0.109, *p* = 0.073) and was significantly negatively associated with working memory at 5 years (PASAT: β = –1.591, *p* = 0.046) and 10 years (β = –0.906, *p* = 0.043).

These findings support IL-8 as a predictor of mid-term processing speed decline and highlight IL-6 as a cytokine with complex, domain-specific cognitive effects in MS. Our data emphasize that baseline inflammatory markers may help identify individuals at risk for later cognitive decline, underscoring the need for larger longitudinal studies.

## 4 Discussion

Our findings identify PPMS-NA as a distinct phenotype within the MS spectrum, defined by low systemic inflammation, central oxidative stress, and glial activation. This neurodegenerative trajectory occurs largely independent of classical lesion activity or peripheral immune responses. This provides the first longitudinal IL-6/IL-8 analysis in PPMS-NA. IL-8 serves as a robust biomarker of future physical disability and cognitive decline, while IL-6 exhibits time- and domain-specific effects on cognitive function. CSF H_2_O_2_ correlates with atrophy, reinforcing oxidative stress as a progression driver. These findings underscore the value of monitoring IL-8 and oxidative stress, supporting early stratification and glia- and mitochondria-targeted therapies, and refining understanding of compartmentalized neuroimmune effects on cognition in MS.

At baseline, IL-6 was lower in PPMS-NA than RRMS, and IFNα2 was reduced across progressive phenotypes, particularly in SPMS, suggesting a dampened type I interferon response. Longitudinally, IL-6 declined less than in RRMS, whereas IL-8 progressively increased, reflecting compartmentalized CNS inflammation in which glial-driven processes occur behind an intact blood–brain barrier and are not fully mirrored in plasma (24, 25). While DMT exposure could theoretically influence cytokine levels, the lack of comparable changes in IL-6 and IFNα2, together with similar longitudinal dynamics between PPMS-NA and PPMS-A, indicates that the elevated IL-8 in PPMS-NA primarily reflects phenotype-specific immune characteristics rather than treatment effects.

Functionally, IL-6 promotes Th17 responses and context-dependent neural effects (26), whereas IL-8, released mainly by astrocytes and microglia, is strongly linked to oxidative stress, mitochondrial dysfunction, and axonal injury, hallmarks of progressive MS pathology (26, 27). Few studies have characterized IL-6/IL-8 dynamics in this MS subtype, making our findings novel (24, 28, 29). The progressive rise in IL-8 thus marks chronic glial activation linking inflammation and neurodegeneration.

Oxidative stress is central to PPMS-NA: CSF H_2_O_2_ levels were higher than RRMS and OND, while PPMS-A showed comparable but not significantly increases, suggesting greater persistent oxidative stress particularly pronounced in the non-active form. This milieu likely drives neurodegeneration via mitochondrial dysfunction, lipid peroxidation, and ROS-driven glial activation, creating a feed-forward loop. The temporal alignment of IL-8 elevation with increased CSF H_2_O_2_, reinforce a glial–oxidative axis in which ROS amplify IL-8 expression through ROS-sensitive pathways, perpetuating local neuroinflammation and tissue damage (30, 31).

MRI markers linked to oxidative stress are closer to neurodegeneration than classical inflammatory lesions. Brain atrophy trended with CSF H_2_O_2_ but not with IL-6, IL-8, or IFNα2; none correlated with Gd-enhancing lesions. Interestingly, IL-8 was inversely associated with baseline T2 lesion load, reflecting subclinical glial-driven inflammation rather than total lesion burden, supporting a model in which PPMS-NA progression is driven by CNS-intrinsic mechanisms rather than relapsing inflammation.

Among biomarkers, plasma IL-8 was the strongest predictor of future physical disability (EDSS ≥6), whereas IL-6 and H_2_O_2_ contributed modestly without improving predictive power, suggesting that persistent subclinical glial activation underlies gradual clinical progression in PPMS-NA.

IL-8 predicted long-term cognitive decline, particularly in processing speed (SDMT), with associations emerging only longitudinally. Elevated baseline IL-8 reflects subclinical neuroinflammation preceding measurable cognitive impairment, consistent with its role in ROS-sensitive pathways involving glial activation, mitochondrial dysfunction, and neuroinflammation independent of age, education, or mood (32, 33).

IL-6 showed a complex, domain- and time-dependent pattern: positively associated with attention at baseline, suggesting an early compensatory role, but longitudinally inversely linked to visuospatial and working memory and attentional decline over ten years. This pattern highlights IL-6’s pleiotropic role, initially adaptive but potentially neurotoxic with chronic exposure and sustained neuroinflammation (34).

Sex differences were minimal. Analyses of key biomarkers, stratified by sex, revealed slightly higher H_2_O_2_ levels in males, but these differences were not significant. Our findings generally reflect consistent cytokine profiles across sexes. This suggests that oxidative and glial mechanisms converge in later MS, possibly due to reduced hormonal protection in females. Overall, these analyses support the robustness of our main findings and emphasize the importance of further investigating sex- and hormone-related factors in progressive MS.

Despite the strengths of our longitudinal, multimodal approach, limitations include small subgroup sizes (PPMS-A, SPMS), lack of quantitative brain volumetry, and independent cognitive and biomarker cohorts, which limit integrated interpretation. Future studies with volumetric MRI and harmonized cognitive protocols in unified cohorts are needed to validate these findings.

Additionally, the limited size of the SPMS subgroup (n=9), prevents firm conclusions about the potential effects of DMT, disease progression, or other subgroup-specific characteristics. Findings in SPMS should therefore be interpreted with caution, and larger, well-characterized SPMS cohorts will be needed in future studies to clarify these associations.

Last, we acknowledge that environmental and lifestyle factors, such as smoking and obesity, can influence circulating cytokine levels and disease progression in MS (35–38). However, detailed longitudinal data on these and other variables (e.g., viral exposures, vitamin D status, physical activity, stress, concurrent medications) were unavailable in our cohort. Furthermore, inclusion of multiple covariates in multivariate models would risk overfitting, given the sample size. Therefore, predictive analyses were restricted to age, sex, and disease duration. Future studies with larger cohorts are needed to assess other potential confounders.

Our findings suggest that PPMS-NA may be a mechanistically distinct progressive MS subtype, marked by low systemic inflammation, elevated oxidative stress, and selective glial activation. IL-8 and CSF H_2_O_2_ consistently predict disability and cognitive decline, reflecting neurodegeneration driven by oxidative stress and glial activity. IL-6 shows domain- and time-specific effects, highlighting the complex role of peripheral immune signals in cognition. These results support therapies targeting oxidative damage, mitochondrial dysfunction, and glial regulation beyond classical immune modulation.

## Data Availability

The raw data supporting the conclusions of this article will be made available by the authors, without undue reservation.
