# Supplementary material for: A glial oxidative signature predicts disability in primary progressive multiple sclerosis and is associated with long-term cognitive decline
Source: Front Immunol. 2026 Apr 27;17:1802339. doi: 10.3389/fimmu.2026.1802339 (PMC13158083; doi:10.3389/fimmu.2026.1802339)
Supplement: Supplementary file 1 [file DataSheet1.pdf]

Supplementary Material

Supplementary Table S1: Diagnostic classification of OND participants by inflammatory profile.

Diagnosis	Number
**Other Neurological Disorders, Non-Inflammatory profile (OND-NI)**	**13**
Migraine	4
Functional neurological disorder	3
Myelopathy	2
Unspecific cephalalgia	2
Vertiginous syndrome	1
Paraparesis	1
**Other Neurological Disorders, Inflammatory profile (OND-I)**	**13**
Myelitis associated with autoimmune disorders (systemic lupus erythematosus, antiphospholipid syndrome)	4
Optic neuritis	3
Idiopathic myelitis	2
Neuromyelitis optica spectrum disorder (NMOSD)	2
Non-MS demyelinating lesions	2

Supplementary Figure S1: Preliminary profiling of basal plasma cytokine and chemokine levels (pg/ml) revealed distinct expression patterns among study groups (RRMS, PPMS-NA, PPMS-A, SPMS, and OND, classified as inflammatory or non-inflammatory). Analytes included (A) GM-CSF, (B) IFNα2, (C) IL-1β, (D) IL-1Ra, (E) IL-6, (F) IL-8, and (G) IP-10, with samples analyzed in duplicate. Results are expressed as mean ± SEM, with *$p < 0.05$ and **$p < 0.01$ indicating significance.

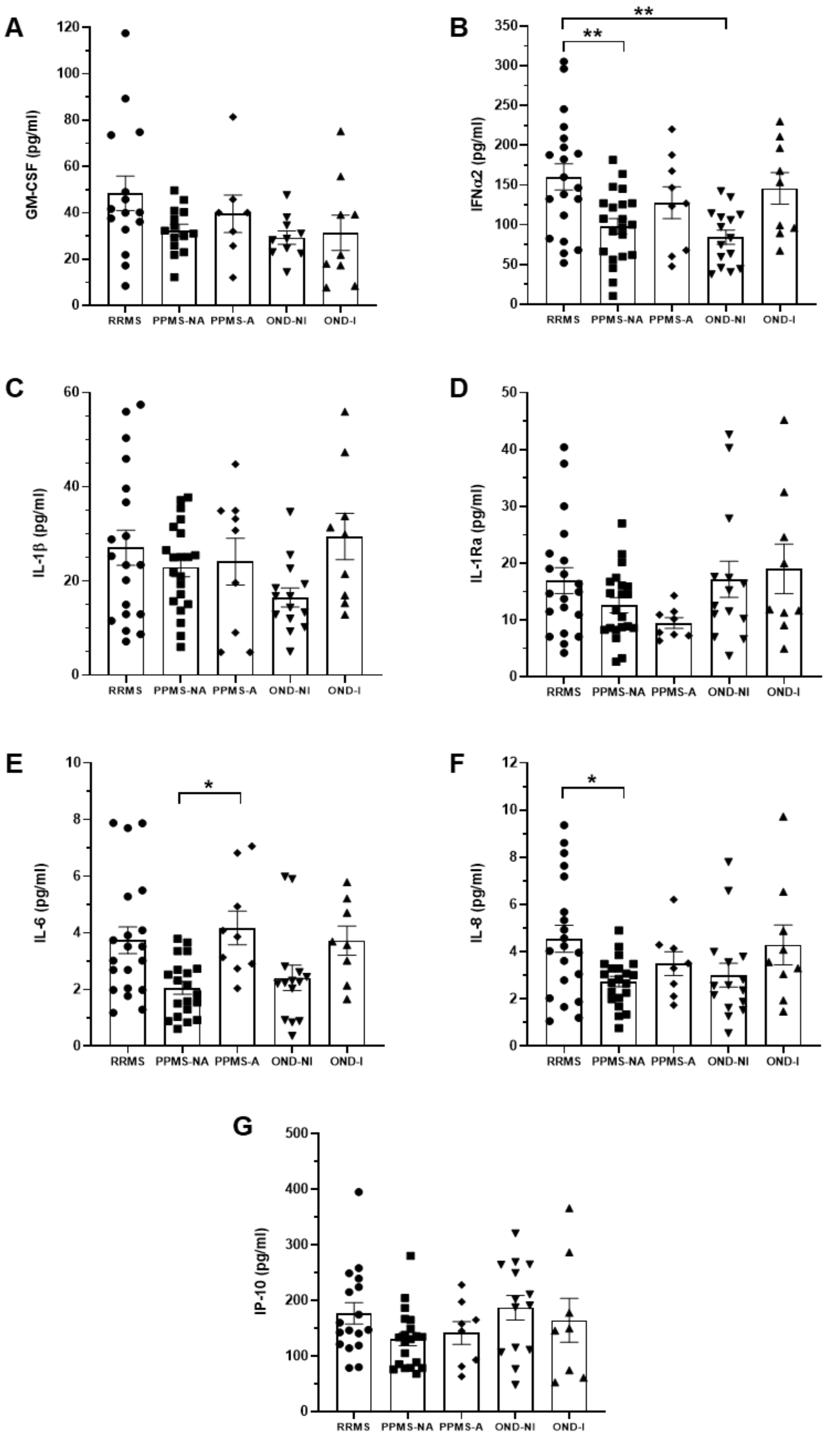

Supplementary Table S2. Sex Differences in H_2O_2, IFNα2, IL-6, and IL-8 at baseline and follow-up. Age was included as a covariate at baseline, and both age and disease duration were included at follow-up. Results are presented as adjusted means (± standard error), F statistics, and corresponding p-values. *$p < 0.05$ and †$p < 0.1$ (trend). n.a.: not available due to insufficient sample size.

Biomarker	Timepoint	Phenotype	Sex	Adjusted Mean (±SE)	F Statistic	p Value
**ROS (H_2O_2)**	Baseline	RRMS	Male<br>Female	48 (9.96)<br>39.7 (10.83)	0.32	0.5784
		PPMS-NA	Male<br>Female	140.35 (29.99)<br>86.73 (25.94)	1.81	0.1948
		PPMS-A	Male<br>Female	59.18 (21.41)<br>51.03 (29.1)	0.04	0.8443
**IFNα2**	Baseline	RRMS	Male<br>Female	111.43 (15.41)<br>137.98 (9.25)	2.11	0.1523
		PPMS-NA	Male<br>Female	91.63 (15.34)<br>108.77 (16.19)	0.58	0.4575
		PPMS-A	Male<br>Female	98.46 (23.75)<br>164.02 (26.89)	3	0.134
	Follow-up	RRMS	Male<br>Female	56.67 (51.62)<br>71.98 (27.68)	84.3	**0.0691†**
		PPMS-NA	Male<br>Female	147.36 (78.16)<br>52.64 (62.98)	0.84	0.5282
		PPMS-A	Male<br>Female	258.02 (44.96)<br>229.02 (18.26)	0.06	0.816
**IL-6**	Baseline	RRMS	Male<br>Female	3.31 (0.53)<br>4.12 (0.37)	1.55	0.2203
		PPMS-NA	Male<br>Female	2.19 (0.37)<br>2.1 (0.4)	0.03	0.8662
		PPMS-A	Male<br>Female	4.28 (1.12)<br>4.17 (1.3)	0	0.952
	Follow-up	RRMS	Male<br>Female	1.66 (0.4)<br>3.47 (0.4)	9.99	**0.0872†**
		PPMS-NA	Male<br>Female	2.82 (0.91)<br>3.75 (1.08)	0.37	0.584
		PPMS-A	Male<br>Female	n.a.<br>4.92 (0.47)	n.a.	n.a.
**IL-8**	Baseline	RRMS	Male<br>Female	4.12 (0.58)<br>5.35 (0.41)	2.88	0.096
		PPMS-NA	Male<br>Female	3.06 (0.27)<br>2.94 (0.27)	0.09	0.765
		PPMS-A	Male<br>Female	3.28 (0.66)<br>4.37 (0.78)	0.96	0.382
	Follow-up	RRMS	Male<br>Female	1.44 (0.28)<br>1.95 (0.32)	1.16	0.331
		PPMS-NA	Male<br>Female	3.92 (1.12)<br>3.52 (0.78)	0.08	0.788
		PPMS-A	Male<br>Female	4.75 (0.88)<br>7.11 (0.52)	91.56	**0.0107***

Supplementary Table S3. Demographic and clinical characteristics of pwMS at baseline and follow-up were included in the cognitive assessment. Data are presented as [1]number (percentage) for categorical variables and [2]median (Q1-Q3) for continuous variables.

**Variable**	**Baseline (Diagnosis)**	**5-year follow-up**	**10-year follow-up**
N	40	37	12
Phenotype[1]			
**CIS**	12 (30)	1 (2.7)	0 (0)
**RR**	28 (70)	35 (94.6)	10 (83.3)
**SP**	0 (0)	1 (2.7)	2 (16.7)
**Sociodemographics**			
Age (years)[2]	41.5 (34.0–45.75)	46.5 (39.0–50.75)	50.0 (43.0–55.0)
Sex[1]			
Female	32 (80%)		
Male	8 (20%)		
Education[1]			
Low	6 (15%)		
Medium	24 (60%)		
High	10 (25%)		

**Clinical and Biomarker Data**			
DMT total[1]			
None	17 (42.5)	1 (2.7)	0 (0)
Moderate efficacy[b]	11 (27.5)	17 (45.9)	4 (33.3)
High efficacy[c]	12 (30.0)	19 (51.4)	8 (66.7)
EDSS[2]	1.5 (0.0–4.0)	3.2 (1.5–5.5)	2.5 (1.5–3.5)
IL-8 (pg/mL)	3.40 (0.55–5.78)	—	—
IL-6 (pg/mL)	4.05 (2.15–5.80)	—	—
Disease duration (years)	0.12 (0.05-0.71)	5.08 (5.02–5.42)	10.23 (10.02–10.95)
